# Current management of juvenile dermatomyositis in Germany and Austria: an online survey of pediatric rheumatologists and pediatric neurologists

**DOI:** 10.1186/s12969-018-0256-7

**Published:** 2018-06-20

**Authors:** Claas H. Hinze, Fabian Speth, Prasad T. Oommen, Johannes-Peter Haas

**Affiliations:** 1 Department of Pediatric Rheumatology and Immunology, University Hospital Münster, Albert-Schweitzer-Campus 1, Building D3, 48149 Münster, Germany; 2 Division of Pediatric Rheumatology, University Medicine, Rostock, Germany; 3 Division of Immunology, Bone Marrow Transplantation and Rheumatology, Ulm, Germany; 4 Center of Child and Adolescent Health, Department of Pediatric Oncology, Hematology and Clinical Immunology, Heinrich-Heine University Duesseldorf, Münster, Germany; 5 German Center for Pediatric and Adolescent Rheumatology, Garmisch-Partenkirchen, Germany

**Keywords:** Dermatomyositis, Surveys and questionnaires, Practice patterns, Physicians, Glucocorticoids, Methotrexate, Antirheumatic agents, Immunoglobulins, Intravenous

## Abstract

**Background:**

Juvenile Dermatomyositis (JDM) is a rare pediatric autoimmune disease with broad variations of the individual course. Data on the optimal management are mostly lacking. Currently treatment decisions are often based on experts’ opinions. In order to develop consensus-based treatment strategies for JDM in Germany a survey was pursued to analyze the current clinical practice.

**Methods:**

An online survey addressing all members of the Society for Pediatric Rheumatology (GKJR) in Germany and Austria and pediatric neurologists with expertise in JDM was performed in February/March of 2016. The questionnaire consisted of 5 case scenarios including diagnostic criteria, treatment of moderate, severe and refractory JDM, using either multiple choice or a 5-point Likert scale. Basic descriptive statistics were used to analyze the findings.

**Results:**

The survey was completed by 60 pediatric rheumatologists and 7 pediatric neurologists experienced in the management of JDM. Typical findings allowing a diagnosis were considered to be: typical skin changes, proximal muscle weakness, MRI findings, elevated muscle enzymes, nailfold capillary changes, presence of calcinosis and muscle biopsy. Regarding induction treatment of moderate/severe JDM: 59%/74% opted for intermittent intravenous methylprednisolone (IVMP) pulse therapy, and 21%/40% for conventional high-dose oral glucocorticoids. Methotrexate (MTX) was the preferred disease-modifying conventional anti-rheumatic drug (cDMARD) for moderate and severe JDM. Regarding the management of refractory moderate or severe JDM, intravenous immune globulins, mycophenolate mofetil and rituximab were preferred treatment options.

**Conclusion:**

There is consensus about the diagnosis of JDM strongly supported by classic clinical and MRI findings. There is great variety in the treatment of JDM in Germany regarding both induction and maintenance therapy. The development of consensus-based treatment strategies for JDM based on harmonization of current clinical practice is essential in order to allow comparative effectiveness research in the future.

**Electronic supplementary material:**

The online version of this article (10.1186/s12969-018-0256-7) contains supplementary material, which is available to authorized users.

## Background

Juvenile dermatomyositis (JDM) is the most common inflammatory myopathy of childhood [1]. Even though it is a rare condition, it is still a major cause of morbidity and mortality among patients with pediatric rheumatic diseases [2, 3]. The Bohan-Peter diagnostic criteria for dermatomyositis (DM) exist for more than 40 years, and those criteria are also often used to diagnose JDM [4]. Several other diagnostic modalities are employed by physicians to diagnose and monitor JDM, including for example, imaging studies and various laboratory markers [5]. Surveys performed in North America demonstrated a high variability in the management of JDM [6]. A European initiative resulted in international consensus-based recommendations and treatment protocols [7, 8]. Currently, it is unclear if practice patterns in Germany vary from those in North America or other countries, so that additional data are desirable.

The PRO-KIND (PROjekte zur Klassifikation, Überwachung und Therapie in der KINDerrheumatologie; projects for the classification, monitoring and therapy in pediatric rheumatology) initiative is a sub-committee of the Society for Pediatric Rheumatology (Gesellschaft für Kinder- und Jugendrheumatologie, GKJR) and aims to define consensus-based protocols to harmonize diagnostic and treatment approaches in Germany. International efforts are currently made to establish disease specific registries for pediatric patients with inflammatory myopathies [9, 10]. However, a German registry sufficiently recording clinical practice and treatment of JDM does not currently exist.

The goal of the PRO-KIND working group on JDM was to identify current practice patterns in Germany via an online survey among pediatric rheumatologists and pediatric neurologists and subsequently to harmonize identified patterns. This manuscript reports on a survey concerning the current practice in diagnosing and managing JDM in Germany.

## Methods

### Study population

The online survey was addressed to all 229 members of the German Society of Pediatric Rheumatology (GKJR) via e-mail with personal invitations to the address available via the society’s membership database. In addition, pediatric neurologists were invited via the website of the German Society for Pediatric Neurology (GNP).

### Instrument

It was estimated that a survey designed to take 30–45 min to complete would be acceptable to participants in the survey. The authors C.H., F.S. and P.O. opted to develop a survey consisting of 5 case scenarios (23 questions) with the option to extend the survey for an additional case scenario addressing the issue of dystrophic calcification (9 questions). The survey specifically addressed (1) initial diagnostic measures taken in a patient with probable JDM, making the diagnosis of moderate JDM and initial treatment steps (10 questions), (2) maintenance treatment in patients with moderate JDM (6 questions), (3) treatment of refractory moderate JDM (3 questions), (4) treatment of glucocorticoid-dependent moderate JDM (1 question), (5) initial treatment of severe JDM and treatment of refractory severe JDM (3 questions), and, optionally, (6) management of dystrophic calcification (9 questions) (Additional file 1). Some of the questions were multiple choice questions, “select all that apply” questions (both with the option to add free text) and some required grading items on a Likert scale. The part of the survey addressing the management of dystrophic calcification eventually was excluded from this manuscript because of the lower number of participants (*n* = 49) in that part of the survey.

### Survey administration

The survey was conducted between February 2, 2016, and March 15, 2016 using the web-based program Survey Monkey (SurveyMonkey Inc.; San Mateo, California, USA; www.surveymonkey.com). A link to the survey was sent out to the pediatric rheumatologists on February 2, 2016, and a link was posted on the Society for Pediatric Neurology’s website on February 15, 2016. A reminder e-mail was sent out to pediatric rheumatologists who had not opened the survey two weeks prior to closing the survey.

### Data on registered patients with JDM in Germany

The National Pediatric Rheumatic Disease Database (“Kerndokumentation rheumakranker Kinder und Jugendlicher”), a registry which annually collects cross-sectional data on pediatric rheumatic disease in Germany, was queried regarding the number of patients with JDM and the number of centers reporting patients with JDM between 2012 and 2016.

### Analysis of responses

Basic descriptive statistics were performed to summarize the responses with Microsoft Excel (Redmond, Washington, USA). Differences in the responses pediatric rheumatologists and neurologists regarding categorical variables were analyzed via chi-square test.

## Results

### Characteristics of the study population

Sixty GKJR members completed the survey for a response rate of 26.2% (60/229), as well as 7 pediatric neurologists (response rate not calculable). According to the National Pediatric Rheumatic Disease Database, 50 pediatric rheumatology centers in Germany have reported patients with JDM to the database between 2012 and 2016 (2012: 77 patients, 2013: 89 patients, 2014: 108 patients, 2015: 121 patients, 2016: 127 patients) of which at least 29 centers, including the top-enrolling centers, were represented within this survey. Of the 60 GKJR members, 57 (85%) were practicing pediatric rheumatologists, and 3 (4%) were either fellows in pediatric rheumatology or general pediatricians with a special interest in JDM. Regarding the type of practice, 30 (45%) worked in a university hospital setting, 31 (46%) in a non-university hospital setting, and 4 (6%) in private practice. All survey participants had treated patients with JDM; two (3%) had treated more than 50, 14 (21%) between 21 and 50, 19 (28%) between 11 and 20, and 31 between 1 and 10 JDM patients. Fifty-eight participants (87%) were currently managing JDM patients.

### Preferred diagnostic tools in patients with probable JDM

Participants were queried to indicated which type of diagnostic tools they would apply in every patient with probable JDM. Diagnostic studies across multiple categories were inquired about, including clinical scores, laboratory testing, imaging studies, apparatus-based studies (e.g. pulmonary function testing), and consultation of other subspecialities (Table 1). The following diagnostics were suggested by at least 75% of participants: Erythrocyte sedimentation rate, glutamate oxaloacetate transaminase (GOT)/aspartate aminotransferase (AST), glutamate pyruvate transaminase (GPT)/alanine aminotransferase (ALT), creatine kinase (CK), lactate dehydrogenase (LDH), antinuclear antibodies (ANA), complete blood count (CBC) with differential count, C-reactive protein (CRP), urea, creatinine, immune globulin (Ig) G/IgA/IgM, electrocardiography (ECG), pulmonary function testing, echocardiography and muscle magnetic resonance imaging (MRI). There were some differences between pediatric rheumatologists and pediatric neurologist concerning the diagnostic testing rendered (chi square *p* < 0.05): Ig levels (pediatric rheumatologists 85% vs. pediatric neurologists 43%), anti-extractable nuclear antigen (ENA) antibodies (78% vs. 29%), capillary microscopy (70% vs. 14%), abdominal ultrasound (65% vs. 0%), serologic testing for infectious agents (42% vs. 0%) and childhood myositis assessment scale (CMAS) testing (83% vs. 0%), and, in contrast manual muscle testing (13% vs. 57%) and muscle biopsy (13% vs. 57%).

**Table 1:** Preferred testing in patients with probable juvenile dermatomyositis

Diagnostic categories	Proportion of participants supporting testing	Specific diagnostic tests
Laboratory diagnostics	> 75% of participants^a^	Erythrocyte sedimentation rate, GOT/AST, GPT/ALT, CK, LDH, ANA, CBC with differential count, CRP, BUN/creatinine, IgG/IgA/IgM
	50–75% of participants^a^	C3 and C4, myositis blot, ENA panel, vWF antigen, total protein, ferritin, thyrotropin, uric acid, aldolase, rheumatoid factor and CCP antibody
	< 50% of participants^a^	Albumin, 25-OH vitamin D, extended myositis blot (including anti-NXP2, -TIF1gamma, −MDA5), serologic testing for certain infections, cardiac-specific troponin, SPEP, NT pro-BNP, fecal calprotectin, serum neopterin, 1,25 (OH)2 vitamin D, tTG-IgA, lymphocyte subpopulations, stool occult blood
Apparatus-based diagnostics or interventions	> 75% of participants^a^	ECG, pulmonary function testing, echocardiography, muscle MRI
	50–75% of participants^a^	muscle ultrasound, nailfold capillaroscopy, abdominal ultrasound
	< 50% of participants^a^	Chest radiograph, EMG, muscle biopsy, swallow study, chest CT
Juvenile dermatomyositis-specific clinical scores or subspecialty consultations	> 75% of participants^a^	N/A
	50–75% of participants^a^	Childhood myositis assessment scale (CMAS)
	< 50% of participants^a^	Disease activity score, dermatology consultation, manual muscle testing (MMT)-8, neurology consultation

*Abbreviations: ANA* antinuclear antibodies, *ALT* alanine aminotransferase, *AST* aspartate aminotransferase, *BUN* blood urea nitrogen, *CBC* complete blood count, *CCP* cyclic citrullinated peptide, *CK* creatine kinase, *CRP* C-reactive protein, *ECG* electrocardiogram, *EMG* electromyogram, *ENA* extractable nuclear antigen, *GOT* glutamate oxaloacetate transaminase, *GPT* glutamate pyruvate transaminase, *Ig* immunoglobulin, *LDH* lactate dehydrogenase, *MRI* magnetic resonance imaging, *NT pro-BNP* N-terminal pro-brain natriuretic peptide, *SPEP* serum protein electrophoresis, *tTG* tissue transglutaminase, *vWF* von Willebrand factor

^a^tests are listed in order of descending frequency

### Establishing a diagnosis of JDM

**Fig. 1:**
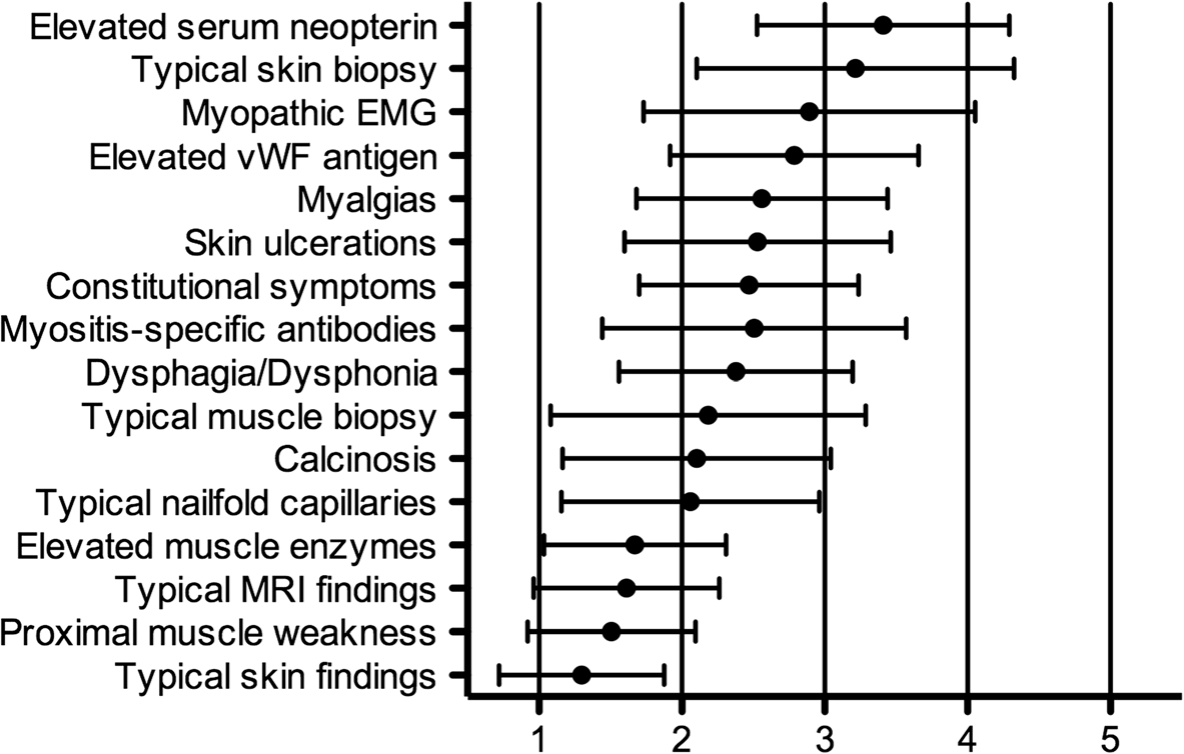
Rating of various findings in establishing a diagnosis of juvenile dermatomyositis. Participants were asked to rate each individual finding in regards to its accuracy in establishing a diagnosis of juvenile dermatomyositis in clinical practice on a 5-point Likert scale (1 = essential, 2 = very important, 3 = somewhat important, 4 = not very important; 5 = not important at all). The mean values +/− standard deviation are given. Abbreviations: EMG, electromyography; MRI, magnetic resonance imaging; vWF, von Willebrand factor

Since clinical symptoms and signs of JDM may vary, participants were asked to rate individual findings regarding their importance in establishing a diagnosis of JDM in clinical practice. Typical skin changes, proximal muscle weakness, typical MRI findings and elevated muscle enzymes were deemed to be most the important features (Fig. 1).

### Experience with validated measures of disease activity or damage

The majority of participants were familiar with several disease activity measures, including the CMAS (91%), the physician global score (87%), the childhood health assessment questionnaire (C-HAQ) (79%), the patient/parent global score (76%), and the disease activity score (DAS) (70%). Less than 50% of participants had experience in using the Pediatric Rheumatology International Trials Organization (PRINTO) core set (40%), the Manual Muscle Testing (MMT) 8-Score (37%), the child health questionnaire (CHQ) (36%), the myositis damage index (MDI) (19%) and the myositis disease activity assessment tool (MDAAT) (13%).

### Initial glucocorticoid therapy in JDM

There was marked variability in the choice of initial glucocorticoid therapy in moderate JDM (Table 2). While in moderate JDM overall 59% of participants opted for intermittent i.v. methylprednisolone pulse (IVMP) therapy, in case of severe JDM, overall 74% opted for intermittent IVMP therapy. High-dose oral therapy (here defined as prednisone equivalent ≥1 mg/kg/day) was chosen in case of moderate JDM by only 21%, but by 40% in case of severe JDM. A large proportion of participants selected options combining intermittent IVMP therapy with either low-dose (prednisone equivalent ≤0.2 mg/kg/day) or moderate-dose (prednisone equivalent > 0.2 to < 1 mg/kg/day) glucocorticoid therapy (38% in moderate JDM and 28% in severe JDM).

**Table 2:** Choice of glucocorticoid regimen in moderate and severe juvenile dermatomyositis

Oral glucocorticoid therapy	Intravenous methylprednisolone pulse therapy					
	Intermittent, ongoing		Once at onset of therapy		None	
	Moderate JDM	Severe JDM	Moderate JDM	Severe JDM	Moderate JDM	Severe JDM
High-dose^a^	5%	28%	6%	10%	10%	2%
Moderate-dose^b^	19%	23%	19%	8%	0%	0%
Low-dose^c^	19%	5%	3%	0%	0%	0%
None	16%	18%	1%	5%	0%	0%

^a^prednisone equivalent of ≥1 mg/kg/day, ^b^prednisone equivalent of > 0.2 to < 1 mg/kg/day, ^c^prednisone equivalent ≤0.2 mg/kg/day

*JDM* juvenile dermatomyositis

### Strategies in glucocorticoid treatment in moderate JDM

More specific patterns on frequency and tapering were defined regarding both IVMP therapy and high-dose oral glucocorticoid therapy. This is an important issue in order to inform a future consensus process (Additional files 1 and 2). For example, the most commonly chosen options for IVMP therapy include a dose of 20 mg/kg body weight for each infusion, administration daily for 3 days every 4 weeks for 6 months. The most commonly chosen options for tapering high-dose oral glucocorticoids include initial taper after 4 weeks, a tapering interval of 4 weeks, reaching moderate-dose levels after 12 weeks, low-dose levels after 6 months and discontinuation after 12 months (Additional file 3).

### Choice of initial conventional disease-modifying antirheumatic drug therapy in JDM

In moderate JDM, 79% opted for starting conventional disease-modifying antirheumatic drug (cDMARD) therapy immediately or within less than 4 weeks after overall initiation of therapy, whereas 4% opted for starting after 8 weeks and 2% for starting after 12 weeks of therapy. Sixteen % opted for not starting any cDMARD if there was a good response to glucocorticoid therapy. Concerning the type of cDMARD chosen, 86% opted for methotrexate (MTX) (37% for oral administration, 49% for subcutaneous administration), 21% for hydroxychloroquin (HCQ), 9% for azathioprin (AZA), 4% for mycophenolate mofetil (MMF) and 1% for cyclosporin A (CSA). Assuming an adequate response to therapy, the preferred duration of MTX therapy was 12 months for 11%, 24 months for 51%, 36 months for 22% and 48 months for 2%.

In severe JDM, 92% of the experts opted for initial therapy with MTX (15% oral, 77% subcutaneous), 62% for intravenous Immunoglobulins (IVIG), 56% for HCQ, 11% for AZA, 10% for rituximab (RTX), 8% for MMF, 7% for CSA, 5% for cyclophosphamide (CYC), 3% for TNF inhibition (TNFi) and 2% for tocilizumab (TCZ).

### Management of refractory and glucocorticoid-dependent JDM

**Fig. 2:**
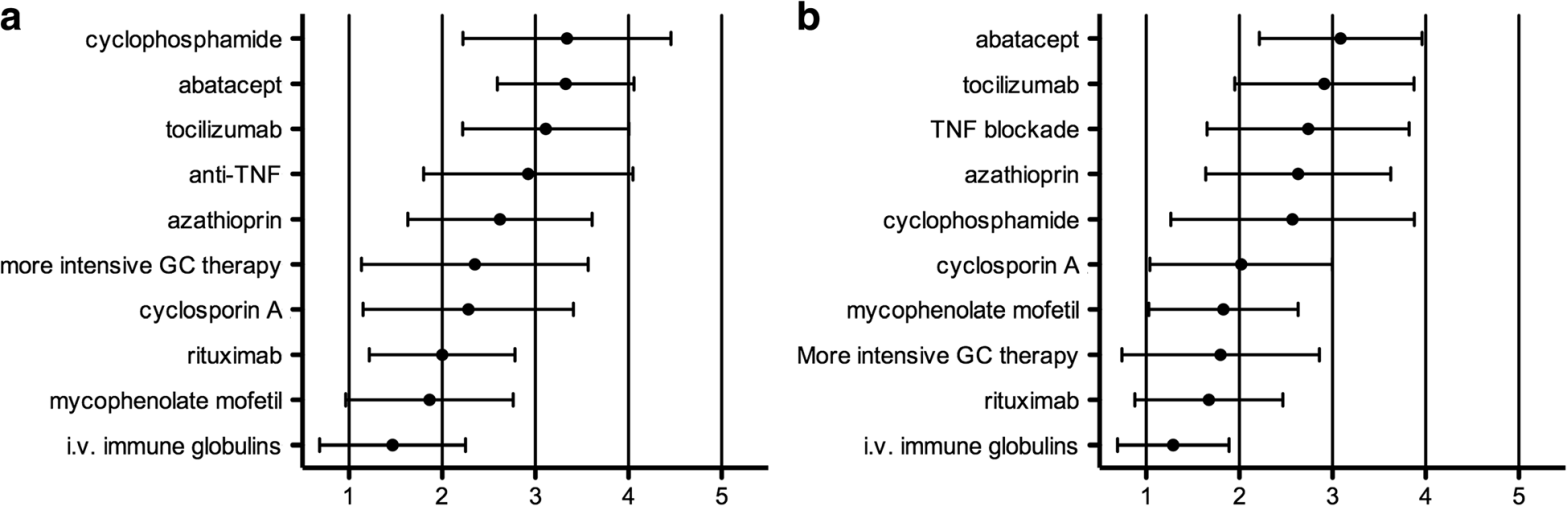
Rating of various treatment options in patients with ongoing refractory (**a**) moderate or (**b**) severe juvenile dermatomyositis on a 5-point Likert scale (1 = support strongly, 2 = support somewhat, 3 = neither support nor reject, 4 = reject somewhat, 5 = reject strongly). Mean and standard deviations are represented

Assuming patients had an inadequate response to therapy after 6 weeks of treatment, 73% suggested an additional therapy, 20% a switch in therapy and 7% no change and longer observation. Of those participants choosing either additional or switch in therapy, IVIG was favored by 67%, repeating or intensifying IVMP therapy by 44%, CSA by 19%, MMF by 14%, AZA by 11%, RTX by 8%, TNFi by 8%, CYC by 2% and TCZ by 2%. Similarly, assuming a glucocorticoid-dependent disease course, i.e. increasing disease activity upon glucocorticoid tapering, 53% suggested an additional therapy, 20% a switch in therapy and 27% a re-intensification of the current therapy. Of those participants choosing an additional or a switch in therapy options were: IVIG (79%), MMF (35%), CSA (31%), AZA (24%), RTX (21%), TNFi (13%), CYC (8%) and TCZ (5%). On a Likert scale of 1 to 5, 1 indicating strong support and 5 strong rejection, the most preferred therapies in ongoing refractory moderate JDM were IVIG, MMF and RTX (Fig. 2a). In case of refractory severe JDM, the preferred options were IVIG, RTX, intensified glucocorticoid therapy and MMF (Fig. 2b). The most common IVIG dosing regimens chosen were 2 g/kg every 4 weeks (by 56%), 1 g/kg every 4 weeks (by 29%) and 2 g/kg every 2 weeks (by 6%). Regarding the time frame expected to observe adequate improvement in severe JDM, the most commonly named time frames were 4 weeks (by 34%), 8 weeks by 21% and 2 weeks by 15%.

## Discussion

Our survey based on case scenarios of patients with typical moderate or severe JDM among pediatric rheumatologists and pediatric neurologist with an expertise in JDM demonstrates marked variability concerning strategies for the diagnosis and management of JDM in Germany. The findings deduced from these analyses will be required for the development of consensus-based treatment strategies in Germany.

An important observation is that the more than 40-year-old Bohan and Peter criteria for the diagnosis of dermatomyositis are still supported by clinical practice [4]. Most experts feel that the demonstration of typical MRI findings should become a valid criterion in the diagnosis of JDM, which is in keeping with other reports [5, 11, 12]. In addition to obtain parameters deemed important to firmly establish the diagnosis JDM, such as MRI, muscle ultrasound, capillary microscopy and myositis-specific antibodies, several additional tests are considered to be informative in order to rule out alternative, overlapping or overarching conditions, to assess for complications of disease and to measure disease activity. As an example, these additional parameters may include serologic testing (e.g. ruling out mimicking infections), autoantibodies (e.g. ruling out lupus and related connective tissue diseases), pulmonary function testing, echocardiography, swallow studies (ruling out severe organ involvement) and/or markers of disease activity. There is evidence on the relevance of myositis-specific antibodies in regard to disease manifestations and disease course, which therefore may impact treatment decisions [13–17]. Severe organ involvement may be present even at the time of diagnosis [18, 19]. Some organ involvement, such as cardiac involvement, may clinically not be apparent, and, thus, justify a screening strategy [20, 21]. The ratings concerning potential markers of disease activity is controversial possibly due to the fact that many of them may be non-specific. However, von Willebrand factor antigen and serum neopterin are currently used by many German Centers [22, 23].

There is marked controversy regarding the treatment of JDM, most prominently concerning the glucocorticoid regimens used. It is well accepted that there is great variability in the use of glucocorticoids in JDM [6, 24]. In comparison to data obtained in a similar survey by CARRA [6], in Germany, intermittent IVMP therapy is preferred much more frequently than in North America, whereas in North America high-dose oral therapy is preferred more frequently. This is also in contrast to current international consensus treatment plans, consensus recommendations and expert opinion that advocate the use of conventional high-dose oral glucocorticoid therapy [1, 7, 8, 25–27]. However, some experts have advocated for the use of intermittent IVMP therapy with the goal to avoid adverse effects of glucocorticoid therapy even though some data point to the fact that IVMP therapy frequently is not sufficiently controlling disease activity [28–31]. Unfortunately, definitive data on the equivalence of conventional high-dose glucocorticoid therapy and IVMP therapy do not exist, even though a randomized trial had been initiated in Germany in the past [32].

As far as the use of additional cDMARD therapy is concerned, there is strong evidence from a rare international randomized controlled clinical trial that MTX and CSA are effective in improving control of disease activity and decreasing the cumulative glucocorticoid exposure [27]. Due to its superior adverse effects profile, the authors of that particularly study recommend methotrexate as first-line therapy over CSA. International recommendations and protocols have firmly established MTX as a standard therapy for moderate JDM [7, 8]. This is also reflected by current clinical practice in Germany as evidenced by this survey, similar to what has been reported in North America [6].

Substantial variability and controversy was identified regarding the treatment of JDM patients with a refractory or severe course of disease. Whereas some experts and treatment protocols place an emphasis on IVIG therapy, others, especially in the United Kingdom, commonly employ CYC therapy, a modality that is rarely used in Germany [7, 8, 33, 34]. Furthermore, while in a randomized placebo-phase controlled clinical trial in patients with refractory adult and juvenile dermatomyositis, RTX had failed to achieve the targeted primary outcome, 83% of patients in that trial achieved the definition of improvement [35]. There is evidence that the JDM subset in that trial performed better than the adult study cohort, e.g. with a two-and-a-half-fold higher chance to achieve the primary outcome [36]. There is very limited evidence for the efficacy of other cDMARDs in JDM, such as MMF [37]. For biologic DMARDS (bDMARD) like TNFi evidence on efficacy or even lack of efficacy is very limited, even though they are frequently used as outlined by a recent survey among CARRA members  [38, 39]. According to our survey, there is also a strong preference for IVIG as a second-line treatment option in patients with refractory or severe JDM, with MMF and RTX both being preferred options. As expected, many practitioners also prefer the time-tested option of a higher intensity glucocorticoid therapy.

While there was substantial experience in the management of JDM, there was a relative lack of experience with formal monitoring tools, indicating that these tools have not permeated into clinical practice in Germany yet. The reason for this is unclear but it should certainly be a goal to use validated tools consistently.

There are several limitations to this work. First of all, the data presented here indicate preferences based on written case scenarios and not real patients. Therefore, these data may not necessary reflect entirely what is happening in clinical practice. Second, out of 229 members of the GKJR, only 60 participated in this survey, so that generalizability may be questioned. However, most patients with JDM are managed in larger pediatric rheumatology centers and those are reflected by our survey. Third, only 7 pediatric neurologists participated in this survey, and, therefore, practice patterns among pediatric neurologists may not be adequately represented by this survey.

## Conclusions

In summary, we have established current clinical practice patterns in Germany in the diagnosis and management of moderate, severe, and refractory JDM based on a survey and case scenarios. Our data demonstrate substantial variability in the management of JDM but common themes emerge. Importantly, the strong preference for using an intermittent IVMP therapy in combination with low or moderate doses of glucocorticoids is in contrast to common practice and recommendations internationally. Methotrexate therapy is considered standard therapy in Germany, as it is internationally. Furthermore, IVIG, MMF and RTX is preferred by many pediatric rheumatologists in Germany in case of severe or refractory JDM. These data are helpful to inform a consensus-based process to establish harmonized strategies for the management of JDM in Germany.

## Additional files

Additional file 1: Case Scenarios used in the online survey. (PDF 123 kb)

Additional file 2: Detailed information on patterns in the use of intravenous methylprednisolone pulse (IVMP) therapy in moderate juvenile dermatomyositis. (A) Doses employed for the individual infusions, (B) the duration of each individual therapy, (C) the overall duration of IVMP therapy and (D) the frequency of IVMP therapy. Abbreviations: IVMP, intravenous methylprednisolone pulse. (JPG 273 kb)

Additional file 3: Detailed information on patterns in the use of high-dose glucocorticoid therapy in moderate juvenile dermatomyositis. (A) Preferred time point of initial glucocorticoid taper also in regards to the presence or absence of improvement, (B) usual interval for glucocorticoid taper, (C) preferred time point for reaching moderate-dose levels, (D) preferred time point for reaching low-dose levels and (E) preferred time point for discontinuation of glucocorticoid therapy. (JPG 374 kb)
