# Supplementary material for: Current management of juvenile dermatomyositis in Germany and Austria: an online survey of pediatric rheumatologists and pediatric neurologists
Source: Pediatr Rheumatol Online J. 2018 Jun 20;16:38. doi: 10.1186/s12969-018-0256-7 (PMC6011340; doi:10.1186/s12969-018-0256-7)
Supplement: Supplementary file 1 — Case Scenarios used in the online survey. (PDF 123 kb) [file 12969_2018_256_MOESM1_ESM.pdf]

Demographic data collected:

- How old are you?
    i. 21-30 Jahre
    ii. 31-40 Jahre
    iii. 41-50 Jahre
    iv. 51-60 Jahre
    v. >60 Jahre
- How would you describe your profession?
    i. Pediatric rheumatologist
    ii. Adult rheumatologist
    iii. Pediatric neurologist
    iv. Dermatologist
    v. Other: ____________
- How would you describe your practice?
    i. Private practice
    ii. University hospital
    iii. Non-university hospital
- Do you treat patients with juvenile dermatomyositis?
    i. Yes
    ii. No
- How many patients with juvenile dermatomyositis have you managed by yourself within your career?
    i. None
    ii. 1-10
    iii. 11-20
    iv. 21-50
    v. >50

***Glossary 1 (could be opened separately)**

Diagnostic criteria for dermatomyositis according to Bohan and Peter (Bohan, A. and J.B. Peter, Polymyositis and dermatomyositis (first of two parts). N Engl J Med, 1975. 292(7): p. 344-347):

1. Sine qua non: Typical dermatologic features (e.g. lilac discoloration of the eyelids (heliotrope) with periorbital edema, a scaly, erythematous dermatitis over the dorsum of the hands (especially the metacarpophalangeal and proximal interphalangeal joints, Gottron's sign), and involvement of the knees, elbows and medial malleoli, as well as the face, neck, and upper torso)

2. Symmetrical weakness of the limb-girdle muscles and anterior neck flexors

3. Muscle-biopsy evidence of necrosis of Type I and II fibers, phagocytosis, regeneration with basophilia, large vesicular sarcolemmal nuclei and prominent nucleoli, atrophy in a perifascicular distribution, variation in fiber size, and an inflammatory exudate, often perivascular

4. Elevation in serum of skeletal-muscle enzymes, particularly creatine phosphokinase and often aldolase, serum glutamate oxaloacetate and pyruvate transaminases, and lactate dehydrogenase

5. Electromyographic triad of short, small, polyphasic motor units, fibrillations, positive sharp waves and insertional irritability, and bizarre, high-frequency repetitive discharges

Definite: three or four criteria (plus the rash)

Probable: two criteria (plus the rash)

Possible: one criterion (plus the rash)

**** Glossary 2 (could be opened separately)**

Patient characteristics according to CARRA (Childhood Arthritis & Rheumatology Research Alliance):

Patients should have:

Rash (Gottron's rash, heliotrope rash, or extensor surface rash)

Muscle weakness

Evidence of myositis (by biopsy, magnetic resonance imaging, or electromyography)

Age <16 years at onset

Physician global assessment of moderate (on a 3-category scale of mild, moderate, or severe)

Patients should NOT have:

Severe disability as defined by can't get out of bed, CMAS score <15, or MMT8 score <30

Parenchymal lung disease

Gastrointestinal vasculitis (as determined by imaging or presence of bloody stools)

Other autoimmune or mimicking disease (as determined by the treating physician)

Requirement for intensive care unit management

Presence of aspiration or dysphagia to the point of inability to swallow

Central nervous system disease (defined as decreased level of consciousness or seizures)

Skin ulceration

Medication contraindication

Myocarditis

Pregnancy

Significant calcinosis (as determined by the treating physician)

Age <1 year

Scenario 1 (moderate JDM, diagnostic testing and induction therapy)

A previously healthy 7-year-old girl presents with progressive malaise of 2 months duration, decreased exercise tolerance, occasional dyspnea on exertion, problems climbing stairs and getting up from the floor, and a rash of the face and extremities. She does not have dysphagia, dysphonia, abdominal pain or hematochezia. On physical exam, you observe a classic heliotrope (lilac discoloration of the eyelids with mild swelling), Gottron sign and papules and periungual erythema. Proximal muscle strength is decreased. For example, she exhibits a positive Gowers maneuver, and getting up from a supine position is not possible. There is mild limitation of movement of the wrist, elbow, hip and knee joints, without evidence for arthritis. Laboratory values 2 weeks ago demonstrated elevated muscle enzymes with a CK of 500 U/l (normal <150 U/l). A complete blood count was within normal limits.

You are concerned about the potential juvenile dermatomyositis (JDM) and determine that a probable JDM is present according to the Bohan and Peter criteria (see glossary) since typical dermatologic features, proximal muscle weakness and elevated muscle enzymes are present.

Question 1

Which of the following testing do you pursue in any case in this situation or perform by yourself (if appropriate)? Select all applicable answers.

1. Physical examination
   - Formal assessment of the Childhood-Myositis-Assessment-Scale (CMAS)
   - Formal assessment via Manual-Muscle-Testing (MMT)
   - Formal assessment of disease activity (e.g. via the Disease Activity Score)
2. Laboratory testing
   - Complete blood count
   - CRP
   - Erythrocyte sedimentation rate
   - GOT (AST)
   - GPT (ALT)
   - LDH
   - Aldolase
   - Creatin-Kinase (CK)
   - Troponin I
   - NT-pro BNP
   - Albumin
   - Total protein
   - Serum protein electrophoresis
   - Elektrolytes
   - Urea, creatinin
   - Uric acid
   - Ferritin
   - Transferrin saturation, iron

- TSH
- Immunoglobulins A, G, M
- Complement C3, C4
- von-Willebrand-Factor (vWF) antigen
- Serum neopterin
- 25-OH-Vitamin D
- 1,25 (OH)2-Vitamin D
- Lymphocyte subpopulations (T-, B-, NK cells)
- ANA
- Anti-ENA panel
- Myositis-specific antibodies via myositis blot (incl. Ro, OJ, EJ, PL-12, PL-7, SRP, Jo-1, PM-Scl75, PM-Scl100, Ku, Mi-2)
- Extended myositis-specific antibodies (incl. Mi-2α, Mi-2ß, TIF1γ, MDA5, NXP2, SRP, Jo-1, PL-7 und 12, EJ, OJ)
- Rheumatoid factor and/or CCP antibodies
- Serologic testing regarding vaccinations or infections (if yes, please list which ones below)
- Transglutaminase-IgA antibodies
- Fecal occult blood
- Fecal calprotectin

3. Apparatus-based studies
   - Muscle biopsy
   - Elektromyography (EMG)
   - Muscle MRI
   - Muscle ultrasound
   - Abdominal ultrasound
   - Echokardiography
   - EKG
   - Pulmonary function testing (Bodyplethysmography)
   - Chest x-ray
   - Chest CT
   - Swallowing study
   - Capillary microscopy
4. Consultations
   - Dermatology
   - Pediatric neurology
5. Other (please name specifically): ____________________

Question 2:

Which of the following findings do you find helpful in order to accurately diagnose typical JDM, i.e. with high sensitivity and specificity in clinical practice? Please rate the findings in regards to making a diagnosis in clinical practice (1 = essential, 2 = very important, 3= somewhat important, 4 = not very important; 5 = not important at all)

Medical history:

- Constitutional symptoms, e.g. malaise or fever
- Myalgia

Physical examination:

- Proximal muscle weakness
- Dysphagia or dysphonia
- Typical dermatologic findings (e.g. heliotrope or Gottron sign)
- Typical nail fold capillary changes
- Skin ulcerations
- Presence of calcinosis/dystrophic calcification

Laboratory testing:

- Elevated muscle enzymes (CK, GOT, LDH, aldolase)
- Elevated vWF antigen
- Elevated serum neopterin
- Presence of myositis-specific or myositis-associated antibodies

Apparatus-based testing:

- Myopathic changes on EMG
- Typical findings on muscle biopsy
- Typical findings on skin biopsy
- Typical findings of muscle inflammation on MRI
- Typical findings on muscle ultrasound

Question 3:

If you pursue MR imaging, what kind of MRI do you pursue? Select all applicable answers.

- MRI of the pelvis
- MRI of the thighs
- MRI of the shoulder girdle
- Whole-body MRI
- Incl. STIR sequences
- Incl. Contrast administration
- Other (please name specficically):________

Question 4:

Which of the following validated tools for measuring JDM are you familiar with? Select all applicable answers.

- Physician global score
- Parent/Patient global score
- Manual Muscle Testing (MMT)8

- Childhood Myositis Assessment Scale (CMAS)
- Disease Activity Score (DAS)
- Myositis Disease Activity Assessment Tool (MDAAT)
- Childhood Health Assessment Questionnaire (C-HAQ)
- Child Health Questionnaire (CHQ)
- PRINTO (Pediatric Rheumatology International Trials Organisation) Core Set
- IMACS (International Myositis Assessment & Clinical Studies Group) Core Set
- Definition of Improvement (DOI)
- Myositis Disease Damage Index (MDI)

Question 5:

You determine that according to Bohan and Peter a definite JDM is present and consider it tob e of moderate severity (see glossary). Which of the following therapies do you initiate immediately or within the first two months of therapy **<u>in any case</u>**? Select all applicable answers.

- i.v. methylprednisolone pulse therapy (≥3 infusions) once
- i.v. methylprednisolone pulse therapy (≥3 infusions) repeatedly
- high-dose oral prednisolone/prednisone therapy (1-2 mg/kg, max. 80 mg daily)
- medium-dose oral prednisolone/prednisone therapy (0.5-1 mg/kg daily)
- low-dose oral prednisolone/prednisone therapy (≤0,2 mg/kg daily)
- oral methotrexate
- subcutaneous methotrexate
- hydroxychloroquin
- azathioprin
- mycophenolate mofetil/mycophenolic acid
- intravenous immune globulins
- rituximab
- vitamin D supplementation
- calcium supplementation
- physiotherapy
- Other (please name specifically): ____________________

Question 6:

In case you pursue repeated i.v. methylprednisolone pulse therapy: How do you specifically apply this therapy (assuming a good response) in regards to dose, frequency and duration? Choose one of the following options.

Dose of each individual infusion (please use the closest dosing):

- Methylprednisolone <10 mg/kg (max. 1000 mg)
- Methylprednisolone 10 mg/kg (max. 1000 mg)
- Methylprednisolone 20 mg/kg (max. 1000 mg)
- Methylprednisolone 30 mg/kg (max. 1000 mg)

Type of individual pulse:

- 1 days
- 2 days in a row
- 3 days in a row

Frequency:

- once weekly
- every 2 weeks
- every 4 weeks

Duration:

- 1 month
- 2 months
- 3 months
- 6 months
- 12 months
- 24 months
- Other (please name specifically): ________________________________

Question 7:

In case you pursue oral prednisolone/prednisone therapy: How do you typically apply this therapy initially? Choose one of the following options.

- Once daily dosing
- Twice daily dosing
- Three times daily dosing
- Other (please name specifically): ________________________________

Question 8:

In case you pursue oral prednisolone/prednisone therapy: When do you start to reduce (taper) the dose? Select all applicable answers.

- After 2 weeks
- After 4 weeks
- After 6 weeks
- After 8 weeks
- When a mild improvement has taken place
- When a major improvement has taken place
- Other (please name specifically): ________________________________

Question 9:

When do you initiate treatment with a steroid-sparing drug? Mutliple choice.

- within ≤ 4 weeks

- after 8 weeks
- after 12 weeks
- in case of good response to steroid therapy not at all
- Other (please name specifically): ________________________________

Question 10:

In case you pursue intravenous immune globulin therapy: What dose and interval are you using? Choose one of the following options.

- 1 g/kg every 2 weeks
- 1 g/kg every 4 weeks
- 2 g/kg every 2 weeks
- 2 g/kg every 4 weeks
- Other (please name specifically): ________________________________

Scenario 2 (moderate JDM, maintenance therapy after >2 months of therapy)

You are managing a 9-year-old patient with moderate JDM. The patient has responded well to induction therapy with high-dose glucocorticoids (either orally and/or as i.v. pulse therapy), methotrexate and hydroxychloroquine within the first 2 months. The following question address treatment beyond the first 2 months.

Question 1:

In case you initially pursued oral prednisolone/prednisone therapy: How do you reduce (taper) the dose? Select all applicable answers.

- Every week
- Every 2 weeks
- Every 4 weeks
- Every 8 weeks
- Initially shorter intervals, later longer intervals
- Initially longer intervals, later shorter intervals
- Other (please name specifically): ________________________________

Question 2:

In case you initially pursued high-dose oral prednisolone/prednisone therapy: When do you plan on reaching a moderately-high dose of 0.5 mg/kg daily? Choose one of the following options.

- After 4 weeks
- After 8 weeks
- After 12 weeks
- After 6 months
- After 12 months
- Other (please name specifically): ________________________________

Question 3:

In case you initially pursued high-dose oral prednisolone/prednisone therapy: When do you plan on reaching a low dose of 0.2 mg/kg daily? Choose one of the following options.

- After 8 weeks
- After 12 weeks
- After 6 months
- After 9 months
- After 12 months
- Other (please name specifically): ________________________________

Question 4:

In case you initially pursued intermittent i.v. pulse methylprednisolone therapy +/- oral glucocorticoids: How often do you administer pulses? Choose one of the following options.

- Once a week
- Every 2 weeks
- Every 3 weeks
- Every 4 weeks
- Other (please name specifically): ________________________________

Question 5:

Assuming that the disease course is favorable: How long do you administer glucocorticoids overall? Choose one of the following options.

- 6 months
- 12 months
- 24 months
- 36 months
- Other (please name specifically): ________________________________

Question 6:

Assuming that the disease course is favorable: How long do you administer methotrexate overall? Choose one of the following options.

- 12 months
- 24 months
- 36 months
- 48 months
- Other (please name specifically): ________________________________

Scenario 3 (Refractory moderate JDM, therapy)

You are managing a 9-year-old girl with moderate JDM, i.e. typical dermatologic features, proximal muscle weakness, elevated muscle enzymes were present but no severe complications. Six weeks ago, you initiated an induction therapy with glucocorticoids (according to your preference) which has not been tapered yet. In addition, methotrexate and hydroxychloroquine were initiated. Clinical symptoms and signs, however, are unchanged. Your clinical judgement is that there is ongoing active moderate JDM and that there has not been adequate improvement. Glucocorticoid adverse effects are present (weight gain, typical cushingoid features). Overall, you consider the disease course refractory.

Question 1:

Which oft he following steps do you pursue? Select all applicable answers.

- Continuation of current therapy for a longer duration to wait for improvement (please name adequate time frame below).
- Continuation of current therapy and addition of another therapy (please check or name below).
- Switch to another therapy (please check or name below).
- Switch to or more intensive i.v. methylprednisolone pulse therapy
- Cyclosporin A (switch or additional therapy)
- Azathioprin (switch or additional therapy)
- Mycophenolate mofetil/mycophenolic acid (switch or additional therapy)
- i.v. immune globulins (switch or additional therapy)
- Cyclophosphamide (switch or additional therapy)
- Rituximab (switch or additional therapy)
- Abatacept (switch or additional therapy)
- TNF blockade (switch or additional therapy)
- Tocilizumab (switch or additional therapy)
- Other (please name specifically): ________________________________

Question 2:

How long should you typically wait after introduction of a new therapy in case of refractory moderate JDM in order to decide if improvement is adequate? Choose one of the following options.

- 1 week
- 2 weeks
- 4 weeks
- 6 weeks
- 8 weeks
- 3 months
- 6 months
- Other (please name specifically): ________________________________

Question 3:

The disease remains refractory. Which medications are useful? Please rate the following medications according to a 5-point Likert scale (1 = support strongly, 2 = support somewhat, 3 = neither support nor reject, 4 = reject somewhat, 5 = reject strongly).

- More intensive glucocorticoid therapy
- Azathioprin
- Cyclosporin
- Mycophenolate mofetil/mycophenolic acid
- i.v. immune globulins (switch or additional therapy)
- Cyclophosphamide (switch or additional therapy)
- Rituximab (switch or additional therapy)
- Abatacept (switch or additional therapy)
- TNF blockade (switch or additional therapy)
- Tocilizumab (switch or additional therapy)
- Other (please name specifically): ________________________________

Scenario 4 (steroid-dependent JDM, therapy)

You determine that a patient with moderate JDM had a good initial response to therapy within the first 2 months of therapy, using high-dose glucocorticoids (either as high-dose oral or i.v. intermittent pulse therapy), methotrexate and hydroxychloroquin. Upon tapering glucocorticoids, there is marked disease flare with increased disease activity affecting skin and muscle. Marked glucocorticoid adverse effects are present. You determine that a steroid-dependent disease course is present.

Question 1:

Which medications are useful? Please rate the following medications according to a 5-point Likert scale (1 = support strongly, 2 = support somewhat, 3 = neither support nor reject, 4 = reject somewhat, 5 = reject strongly).

- More intensive glucocorticoid therapy
- Azathioprin
- Cyclosporin
- Mycophenolate mofetil/mycophenolic acid
- i.v. immune globulins (switch or additional therapy)
- Cyclophosphamide (switch or additional therapy)
- Rituximab (switch or additional therapy)
- Abatacept (switch or additional therapy)
- TNF blockade (switch or additional therapy)
- Tocilizumab (switch or additional therapy)
- Other (please name specifically): ________________________________

Scenario 5 (severe JDM, therapy)

A 6-year-old girl presents with complaints of 2 months duration. A definitive JDM is present. Your exam demonstrates a Childhood Myositis Assessment Scale (CMAS) score of 10 (out of max. 52) and the patient is bedridden. She also has dysphagia, abdominal pain, bloody diarrhea, anasarca, cutaneous ulcerations without dystrophic calcifications. She has not received any treatment yet.

Please assume that the complaints/findings are due to JDM disease activity. You determine that a severe JDM is present.

Question 1:

Which of the following therapies do you initiate in any case? Select all applicable answers.

- i.v. methylprednisolone pulse therapy (≥3 infusions) once
- i.v. methylprednisolone pulse therapy (≥3 infusions) repeatedly
- high-dose oral prednisolone/prednisone therapy (1-2 mg/kg, max. 80 mg daily)
- medium-dose oral prednisolone/prednisone therapy (0.5-1 mg/kg daily)
- low-dose oral prednisolone/prednisone therapy (≤0,2 mg/kg daily)
- oral methotrexate
- subcutaneous methotrexate
- hydroxychloroquin
- azathioprin
- mycophenolate mofetil/mycophenolic acid
- cyclophosphamide
- intravenous immune globulins
- rituximab
- abatacept
- TNF blockade
- Tocilizumab
- vitamin D supplementation
- calcium supplementation
- physiotherapy
- Other (please name specifically): ____________________

Question 2:

The disease course is refractory. Which medications are useful? Please rate the following medications according to a 5-point Likert scale (1 = support strongly, 2 = support somewhat, 3 = neither support nor reject, 4 = reject somewhat, 5 = reject strongly).

- More intensive glucocorticoid therapy
- Azathioprin
- Cyclosporin
- Mycophenolate mofetil/mycophenolic acid
- i.v. immune globulins (switch or additional therapy)

- Cyclophosphamide (switch or additional therapy)
- Rituximab (switch or additional therapy)
- Abatacept (switch or additional therapy)
- TNF blockade (switch or additional therapy)
- Tocilizumab (switch or additional therapy)
- Other (please name specifically): ________________________________

Question 3:

How long should you typically wait after introduction of a new therapy in case of refractory severe JDM in order to decide if improvement is adequate? Choose one of the following options.

- 1 week
- 2 weeks
- 4 weeks
- 6 weeks
- 8 weeks
- 3 months
- 6 months
- Other (please name specifically): ________________________________

Scenario Calcinosis (diagnostic testing and therapy):

Question 1:

How do you determine the presence or absence of calcinosis in newly diagnosed patients with JDM? Select all applicable answers.

- Medical history
- Physical examination
- Laboratory testing
- Imaging studies
- No formal testing for calcinosis
- Other (please name specifically): ______________________

Question 2:

If you suspect that calcinosis is present, which imaging or laboratory studies do you typically pursue? Select all applicable answers.

- No additional studies
- Ultrasound
- X-ray
- CT
- MRI
- Szintigraphy
- Serum calcium
- Serum phosphate
- Urine calcium
- Parathormone
- Vitamin D
- Additional studies to determine disease activity
- Other (please name specifically): ______________________

Question 3:

If calcinosis is present, do you classify the type of calcinosis, e.g. subcutaneous nodular, tumoral, fascial, exoskeleton? If yes, why?

- No
- Yes (please explain): ____________________

Question 4:

In which scenario to you typically consider a directed therapy of calcinosis? Select all applicable answers.

- Active JDM with new calcinosis
- Active JDM with persistent calcinosis
- Inactive JDM with new calcinosis
- Inactive JDM with persistent calcinosis
- Other (please name specifically): _______________________

Question 5:

For which type of calcinosis (if it is relevant for you) do you typically consider a specific therapy, independent of JDM activity? Select all applicable answers.

- Subcutaneous nodular
- Tumorous
- Fascial
- Exoskeleton
- Other (please name specifically): _______________________

Question 6:

Please rate how important the following factors are to decide on a targeted therapy of calcinosis on 5 5-point Likert scale (1 = essential, 2 = very important, 3 = somewhat important, 4 = not very important, 5 = not at all important)

- A certain calcinosis type
- Pain
- Limitation of movement
- Surrounding structures are threatened
- Chronic or recurrent superinfection
- Cosmetic factors
- Certain myositis-specific antibodies
- Other signs of active JDM
- Other (please name specifically): _______________________

Question 7:

Which therapy do you typically pursue in case of new calcinosis (your measure of first choice)? Select all applicable answers.

- None specifically, except for therapies that are adequate for the JDM disease activity at the time, i.e. very good control of JDM disease activity is of paramount importance
- Intensification of JDM therapy, i.e. intensification of immunosuppressive or immunomodulatory therapy
- Systemic anti-inflammatory therapy
- Local therapy (e.g. glucocorticoid injection)
- Surgical removal
- Lithotripsy
- Medications affecting calcium and/or phosphate metabolism

Question 8:

In case you have ever used therapies specifically targeting calcinosis in a patient with JDM: Which therapies have you applied? Select all applicable answers.

- Aluminum hydroxide
- Probenecid
- Bisphosphonates
- Diltiazem
- Sodium thiosulfate
- Systemic glucocorticoids
- Local glucocorticoids
- Methotrexate
- Cyclosporin
- i.v. immune globulins
- Mycophenolate mofetil/mycophenolic acid
- Colchicine
- Thalidomide
- Rituximab
- Abatacept
- TNF blockade
- Tocilizumab
- Antibiotic therapy
- Warfarin/coumadin
- Surgical removal
- Lithotripsy
- Other (please name specifically): ______________________

Question 9:

Which therapies, in your experience, are potentially effective in the specific therapy of calcinosis? Select all applicable answers.

- There does not seem to be a specifically effective therapy.
- Aluminum hydroxide
- Probenecid
- Bisphosphonates
- Diltiazem
- Sodium thiosulfate
- Systemic glucocorticoids
- Local glucocorticoids
- Methotrexate
- Cyclosporin
- i.v. immune globulins
- Mycophenolate mofetil/mycophenolic acid

- Colchicine
- Thalidomide
- Rituximab
- Abatacept
- TNF blockade
- Tocilizumab
- Antibiotic therapy
- Warfarin/coumadin
- Surgical removal
- Lithotripsy
- Other (please name specifically): ______________________
