# Supplementary figures and images for: Current management of juvenile dermatomyositis in Germany and Austria: an online survey of pediatric rheumatologists and pediatric neurologists
Source: Pediatr Rheumatol Online J. 2018 Jun 20;16:38. doi: 10.1186/s12969-018-0256-7 (PMC6011340; doi:10.1186/s12969-018-0256-7)

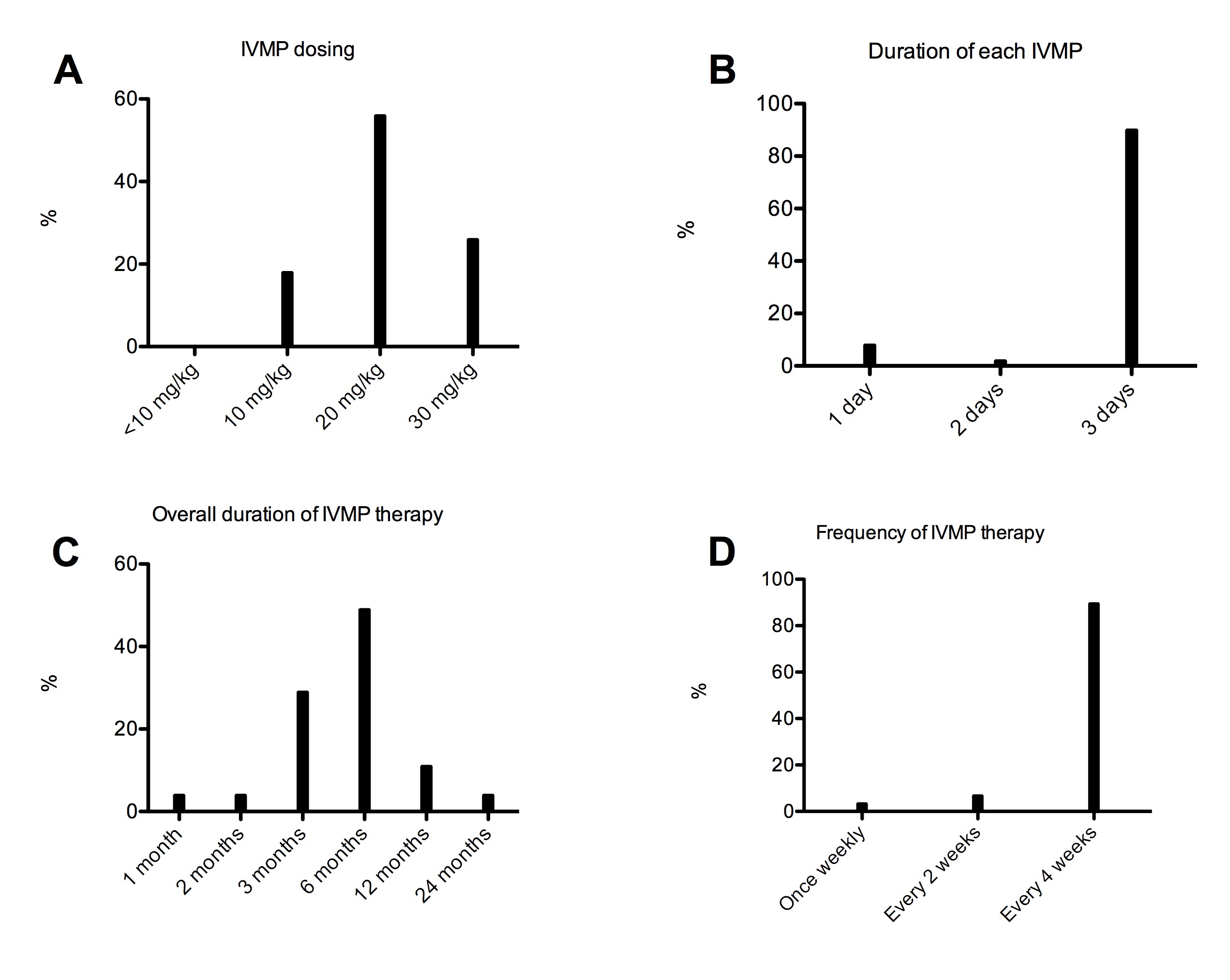

Supplement: Supplementary file 2 — Detailed information on patterns in the use of intravenous methylprednisolone pulse (IVMP) therapy in moderate juvenile dermatomyositis. (A) Doses employed for the individual infusions, (B) the duration of each individual therapy, (C) the overall duration of IVMP therapy and (D) the frequency of IVMP therapy. Abbreviations: IVMP, intravenous methylprednisolone pulse. (JPG 273 kb) [file 12969_2018_256_MOESM2_ESM.jpg]

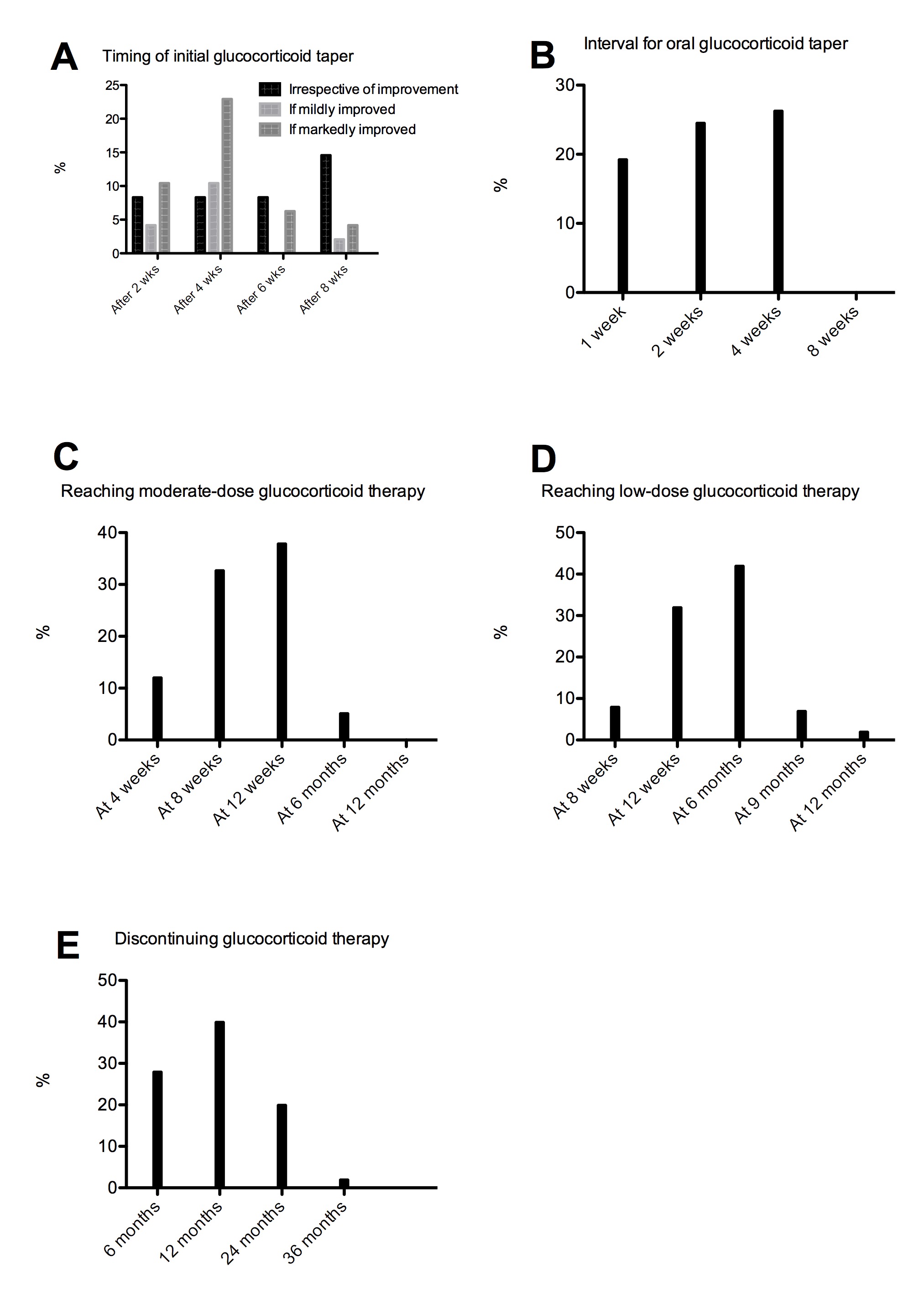

Supplement: Supplementary file 3 — Detailed information on patterns in the use of high-dose glucocorticoid therapy in moderate juvenile dermatomyositis. (A) Preferred time point of initial glucocorticoid taper also in regards to the presence or absence of improvement, (B) usual interval for glucocorticoid taper, (C) preferred time point for reaching moderate-dose levels, (D) preferred time point for reaching low-dose levels and (E) preferred time point for discontinuation of glucocorticoid therapy. (JPG 374 kb) [file 12969_2018_256_MOESM3_ESM.jpg]
